# Barriers to positive bystander behavior: understanding undergraduate physical education students’ attitudes and intentional behavior in gender-based violence prevention

**DOI:** 10.3389/fspor.2025.1569307

**Published:** 2025-07-14

**Authors:** Tiphaine Clerincx, Hebe Schaillée, Inge Derom

**Affiliations:** Research Unit Sport & Society, Department of Movement and Sport Sciences, Faculty of Physical Education and Physiotherapy, Vrije Universiteit Brussel, Brussels, Belgium

**Keywords:** gender-based violence, higher education, physical education, sports, bystander behavior

## Abstract

**Introduction:**

Gender-based violence (GBV) is a global issue that is reported in multiple contexts, including higher education institutes (HEI) and sports settings. Individuals whose gender identity or gender expression diverges from the dominant norms within sports or HEIs are at an increased risk of experiencing GBV. Gender-based violence is broadly defined as any form of interpersonal violence directed at individuals based on their gender identity or gender expression. Undergraduate physical education (PE) students are uniquely positioned to act as change agents in the prevention of GBV, given their connection to both education and their ambition to become professionals in the sport sector. The aim of this study is to investigate PE students’ perceptions of GBV, their attitudes, and intentional bystander behavior when witnessing GBV.

**Methods:**

An explorative qualitative research design using focus groups was conducted in Spain and Belgium and involved 65 undergraduate PE students selected through convenience sampling from 5 HEIs, 41 identified as males and 24 identified as females. Data were gathered through 9 focus groups guided by a semi-structured interview guide and subsequently analyzed via Nvivo through reflective thematic analysis.

**Results:**

Students perceived GBV as psychological, physical, and sexual violence directed at individuals based on their sex or gender identity. Their perceptions of GBV prevention were shaped by social norms and generational differences. Key barriers to intervening in GBV situations included societal norms, a lack of competencies, and the natural stress response PE students experienced when witnessing GBV.

**Discussion:**

The findings of this study underscore the need to shift GBV prevention interventions from merely raising awareness and disseminating knowledge to equipping PE students with skills in stress management and self-regulation. These insights lay the groundwork for developing more effective, context-sensitive GBV prevention interventions within HEIs.

## 1 Introduction

Interpersonal violence has a high prevalence rate in sports and on university campuses (1–4). When looking at the prevalence rate of interpersonal violence research shows that girls and women, lesbian, gay, bisexual, transgender, queer, intersex, asexual, and more (LGBTQIA+) individuals are at a disproportionally higher risk of experiencing sexual violence then men (5, 6). One study found that 47.7% of female college students experienced unwanted sexual contact during their first year of enrollment (7). Another study reported that students of the LGBTQIA+ community and women with a more masculine gender expression were at a higher risk of experiencing sexual violence compared to men or heterosexual students (8). The same was found in sports, LGBT athletes have a higher risk of experiencing interpersonal violence compared to heterosexual athletes (5, 9).

These findings illustrate that individuals with a sexual orientation, gender identity or gender expression that differs from the prevailing gender or sexuality norm within the context of sports or university experience disproportionally more interpersonal violence in comparison to individuals that do match the prevailing gender or sexuality norm of the context. Thus, suggesting a high prevalence of GBV in sports and university. Gender-based violence is described as psychological, physical, sexual, or neglectful violence directed at an individual's biological sex, gender identity, gender expression, or perceived conformity to gender norms (10). This definition, which includes arbitrary deprivation of liberty and economic deprivation in both public and private life, reflects a progressive understanding of gender by moving beyond the binary distinction between men and women.

In response to the high prevalence of interpersonal violence, various prevention interventions have been developed, primarily school-based programs aimed at increasing GBV-related knowledge among children and adolescents (11–13). Interventions targeting college students typically focus on sexual violence prevention but show limited effectiveness (14). Moreover, GBV prevention efforts tailored to undergraduate students remain scarce. This gap has been identified among social work and psychology students, revealing insufficient competences to address GBV and underscoring the potential of targeted prevention programs to enhance students' awareness, knowledge, and skills (15).

Given the limited effectiveness and availability of GBV prevention interventions for undergraduates (14, 15), PE students remain an overlooked yet critical group. As future professionals in sport-related roles, such as teachers, coaches, or policymakers, they are well positioned to promote safe environments. Equipping them with the necessary skills to address GBV is therefore essential. This study examines PE students' knowledge, attitudes, and intended behaviors related to GBV prevention.

To be able to educate PE students on fostering a safe and inclusive sport climate and positive intervene in GBV situations, it is necessary to understand which aspects influence positive proactive and reactive bystander behaviors (16). Two key frameworks inform this understanding: the bystander model (17) and the theory of planned behavior (18). These models highlight both intrapersonal, such as knowledge, attitudes, and interpersonal, such as subjective norms, determinants of behavior. For instance, increased GBV-related knowledge has been shown to raise risk awareness and improve recognition of potentially harmful situations (19). However, knowledge alone is insufficient; according to the bystander model, individuals must first perceive a situation as problematic before they can act (17).

The theory of planned behavior further outlines three determinants of intention: positive attitudes toward prevention, alignment with social norms, and perceived behavioral control (18). Together, these factors influence whether bystanders choose to intervene. Accordingly, GBV prevention efforts targeting undergraduate PE students should not only increase knowledge but also foster supportive attitudes, challenge harmful norms, and enhance students' confidence in their ability to act. Addressing these dimensions can better prepare future sport professionals to adopt proactive and reactive bystander behaviors—contributing to more responsive and inclusive sport settings.

In this study focus groups were conducted with undergraduate PE students in higher education institutions, to increase our understanding on students' knowledge, attitudes, and intentional behavior regarding GBV.

## 2 Method

### 2.1 Research context

This study is part of a larger European co-funded project named The Transformative Power of Sport for and by Students that aimed to develop, implement, and evaluate a GBV prevention intervention for PE students. The intervention mapping approach (20) was used to conduct the three different phases of the project. The first step of the intervention mapping approach, the need assessment, consisted of 9 focus groups with undergraduate PE students, enrolled in 5 HEIs in Spain and Belgium.

### 2.2 Research design

An explorative qualitative research design was used to uncover the knowledge, attitude, and intentional behavior regarding the prevention of GBV in sports of Belgian and Spanish undergraduate PE students. This was achieved by conducting focus groups with undergraduate PE students studying at one of the five HEIs (Vrije Universiteit Brussel, Erasmushogeschool Brussel, Univeristé de Liège, Universitad de Sevilla, and Universitad de Valencia). The 5 HEIs were selected through convenience sampling. All HEIs were informed about the aim of the EU co-funded project before their participation in this study. Once the HEIs agreed to participate in the project, the HEIs selected one staff member to facilitate the recruitment of the undergraduate PE students for the focus groups. All HEIs were free to choose to which undergraduate PE year they would disseminate the information related to the focus groups. The undergraduate PE students were recruited through purposive sampling. They were informed about the purpose of the study by the selected staff member of their HEI. The PE students who choose to participate in the focus groups could register via a physical or online list that was distributed by the selected staff member of the HEI. All HEIs received the materials via Word document and Microsoft form weblink to distribute to the PE students.

### 2.3 Data collection

The focus groups were conducted on the respective campuses of the HEIs in the language spoken at the HEI. The participation in the focus group was on a voluntary basis. The HEIs could provide an incentive to the participating PE students to thank them for their involvement. The focus groups were moderated by one of the project consortium members who received a detailed protocol and a training on how to conduct the focus groups. Furthermore, one observer joined the moderator for each focus group to support the credibility of the data collection (21). The observer was asked to note whether the data procedure and semi-structured interview guide were respected.

A total of 9 focus group, excluding the pilot study, were conducted across 5 HEIs. 6 focus groups were held in Belgium, each comprising a minimum of 6 and a maximum of 8 PE students. In Spain, 3 focus groups were conducted, ranging from 7 to 12 PE students per focus group.

Before the start of each focus group, participants were asked to complete an online survey via Qualtrics XM to map the demographics of the study population. This was done to provide necessary information on the study population. PE students had the option, for sensitive questions such as, sexuality or migration background to select “I do not want to answer”. Additionally, for gender and sexuality questions, we provided a comprehensive range of potential answers to be as inclusive as possible. The online survey was completed at the start of the focus group, following informed consent from the PE students. Given the sensitive nature of the topic, informed consent was explained verbally to ensure full understanding, therefore no data collection was done in advance.

A semi-structured interview guide was developed based on the theory of planned behavior (18) and the stages of bystander behavior (17). As the aim of the focus groups was to inquire about the knowledge, attitudes, and intentional behavior of PE students regarding GBV, we formulated three themes: perceptions of GBV, including when a situation is recognized as GBV; attitudes and intentional behavior as bystanders, such as potential actions if witnessing GBV and emotional responses to such situations; and leadership responsibility, referring to the perceived role of individuals in leadership positions in preventing GBV. To encourage participants to discuss their bystanders' attitudes and intentional behavior, four cases of GBV were presented —two involving verbal violence as mild forms and one each involving sexual and physical violence as severe forms.

The interview guide was initially developed in Dutch, translated in French, and subsequently back translated from French to Dutch. All anomalous translations were discussed between the two persons involved in the back translation. The Spanish interview guide was translated from Dutch to English and then translated into Spanish. The Spanish version has not been subjected to a back translation due to a lack of Spanish language skills among research team. The Dutch interview guide was tested in one pilot focus group involving second-year undergraduate PE students (*n* = 6) of the HEI (Vrije Universiteit Brussel). The pilot focus group resulted in one change related to leadership responsibility, which was incorporated into the French and Spanish interview guides. This led to the final interview guide.

Due to the sensitivity of the topic, and study participants possible hesitation to express their thoughts on the subject. It was important to lead the focus groups with a shared-power and non-coercive approach (21). Physical education students were given ample time to express their feelings and thoughts. They were also allowed to leave the focus group at any time, and breaks could be integrated upon request. All PE students received a list of main helplines in their respective countries. Additionally, the moderator and observer were physically present for 10 min after the completion of the focus group, in case any PE student wished to disclose further information. Prior to conducting the focus groups, all moderators and observers were provided with a protocol and referral document to guide them in the event a PE student disclosed experiences of violence.

### 2.4 Participants

A total of 69 undergraduate PE students, born between 2000 and 2005, participated in the focus groups. Four PE students from Spain agreed to participate in the focus groups but did not consent to share demographic information. Therefore, the descriptive statistics cover data of only 65 PE students (Table 1). Most participating PE students identified as male (*n* = 41), heterosexual (*n* = 58), and had no migration background (*n* = 47). Many participating PE students had experiences in leadership roles (*n* = 54). Most participants were first-year undergraduate PE students (*n* = 40), and 38 had not yet completed a course on ethical issues where GBV had been discussed. The sample in this study does not primarily consist of individuals who report being at higher risk of experiencing GBV in sports. As a result, the perspectives on GBV prevention are largely shaped by students who may be perceived as conforming to standard gender norms.

**Table 1:** Descriptive information of study participants.

Demographics	Belgium	Spain	Total	
	*n* = 40	*n* = 25	*N* = 65	100%
Gender				
Female	15	9	24	37
Male	25	16	41	63
Sexuality				
Heterosexual	37	21	58	89
Lesbian	1	2	3	5
Bisexual	2	2	4	6
Migration background				
Yes	16	2	18	28
No	24	23	47	72
Had a leadership function				
Yes	34	20	54	83
No	6	5	11	17
Undergraduate year				
1	18	22	40	62
2	8	3	11	17
3	14		14	21
Followed a course on ethical issues				
Yes	8	13	21	32
No	26	12	38	58
I do not know	6		6	9

### 2.5 Data analysis

The focus groups were audio recorded and transcribed verbatim by the moderator of the focus groups. The focus groups conducted in Dutch and French were transcribed and analyzed in their original languages. The focus groups conducted in Spanish were transcribed in Spanish and translated into English for analysis. The denaturalism approach was used during the transcription process. During the data analysis all idiosyncratic elements of speech such as, stutters, pauses, nonverbals, involuntary vocalizations) were removed (22) as the aim of the study was to analyze the perceptions of undergraduate PE students on the prevention of GBV. All transcripts were pseudonymized.

The focus group transcripts were deductively and inductively analyzed via NVivo software using the reflexive thematic approach (23). This approach enabled a deeper comprehension of how undergraduate PE students understand the concept of GBV and their predisposition on the prevention of GBV in the sport and university context. The first author conducted the reflective thematic analysis starting from her own comprehension of the theoretical framework used to construct the semi-structured interview guide, including the theory of planned behavior (18) and stages of bystander behavior (17). This subjective reflection on the dataset drove the first exploration of the data, followed by a first data coding and the creation of initial themes. Theoretical data triangulation (21) was applied during the data analysis. The proactive and reactive bystander matrix (16) and the window of tolerance (24) theories were added during the coding process, thus providing a holistic theoretical perspective on the data and increase its validity. The data analysis was not a linear process. The researcher went back and forth in creating themes and assessed the obtained information of the PE students to the already existing theories and their own subjective beliefs, with the aim of introducing all relevant nuances related to the perceptions, attitudes and intentional behavior of the study participants and their needs related to the prevention of GBV. Excerpts of the focus groups have been added in the results sections to provide evidence of data patterns.

### 2.6 Trustworthiness

Trustworthiness was ensured through careful attention to credibility, transferability, dependability, and confirmability. The credibility was supported by using a theory-informed semi-structured interview guide as well as protocols for moderators and observers, which helped to ensure that the data collected were consistent across the different HEIs. Including participant quotes in the results further grounded the findings in the lived experiences of the participants, increasing confidence in their authenticity. The transferability was supported through detailed contextual and demographic descriptions. The study included undergraduate PE students from five HEIs across Belgium and Spain, providing a degree of institutional and cultural diversity. However, as the HEIs were selected through convenience sampling and participants self-selected into the focus groups, the sample may not fully represent all PE students, particularly those at higher risk of experiencing GBV. The dependability was reinforced by clearly documenting research procedures, the use of NVivo software for systematic coding, and an inductive and deductive approach to data analysis. The confirmability was promoted through reflexive thematic analysis, acknowledgment of the researcher's positionality, theoretical triangulation, and pseudonymization of data. Additionally, ethical considerations such as voluntary participation, the option to skip sensitive questions, and post-session support contributed to the authenticity of the findings by ensuring a respectful and inclusive research environment (21).

## 3 Results

Four main themes with theoretical underpinning were identified from the analysis of the focus groups. The first two themes focus on knowledge and attitudes of students on GBV, whereas the second two themes address positive proactive and reactive bystander behavior. The results section will explain the four identified themes in detail: (1) PE students' perception of GBV in sports and HEIs, (2) PE students' attitudes towards (the prevention of) GBV, (3) strategies employed by PE students when acting as bystanders during incidents of GBV, and (4) perceived barriers influencing student's positive reactive bystander behavior.

### 3.1 PE students' perception of GBV in sports and HEIs

Two main perceptions of GBV were identified: a progressive view and a binary view. A clear cultural difference was observed between Belgian and Spanish PE students. Most Belgian PE students adopted a progressive view, defining GBV as psychological, physical, or sexual violence based on a person's sex or gender identity (e.g., male, female, non-binary, transgender). They identified various forms of GBV such as sexism, homophobia, partner violence, and discrimination and referred to criteria like power imbalance, intentionality, repetition, and severity. Notably, neglect was not mentioned by participants.

In contrast, most Spanish PE students expressed a binary understanding of GBV, viewing it primarily as male violence against women, rooted in beliefs of male superiority. They did not recognize violence against men as GBV and attributed violence against women to be entrenched in gender hierarchies and power dynamics.

For me, violence—whether physical or psychological—is any act a man inflicts upon a woman. If the roles were reversed, it would be considered something else and not classified as gender-based violence (PE student Spain).

In general, PE students perceived GBV as context-dependent, shaped by temporal and cultural factors. They indicated that whether a situation is identified as GBV may vary according to prevailing social norms and generational perspectives. For instance, some participants stated that if an older professor failed to use a student's correct pronouns, they might excuse that behavior, attributing it to generational differences rather than intentional harm.

I think our generation pays more attention to these things [GBV situation]. We are more aware of issues like sexually inappropriate behavior or actions that are unacceptable compared to the generations before us. I believe we are much more engaged with these topics. This is also influenced by (social) media and smartphones. We are much more conscious of the consequences (PE student Belgium).

### 3.2 PE students' attitudes towards (the prevention of) GBV

An unexpected finding during the focus groups was that all PE students disclosed having witnessed at least one GBV situation in their lifetime. Their reflections on GBV were shaped by personal experiences, either as bystanders or victims. These experiences elicited strong emotional responses, including anger, fear, hurt, helplessness, and a sense of injustice. Students unanimously identified scenarios such as a boy being assaulted for kissing his boyfriend or a boy touching a girl without consent in a bar as clear instances of GBV. Situations involving physical contact consistently provoked negative reactions, with all participants deeming such acts unacceptable.

While most attitudes toward GBV were clearly condemnatory, two situations prompted more nuanced views. The first involved sexist or gender-related jokes. Some participants believed humor should be permitted among peers, if the person making the joke understands the group dynamic, anticipates a positive reception, and is prepared to apologize if it is perceived offensive. The general consensus, however, was that if there is uncertainty about how a joke might be received, it is best not to make it. All students agreed that individuals in positions of authority—such as professors or coaches—should avoid jokes related to sex, gender identity, or expression. The power imbalance inherent in these roles renders such humor inappropriate. PE students emphasized the role of academic and sport staff as role models, responsible for setting norms and shaping what is considered acceptable conduct.

Personally, I think I would not do it [make a sexist or gender joke] because, in any case, we do not know the impact it could have on the person the joke is aimed at (PE student Belgium).

The second situation that elicited ambivalent attitudes was primarily noted among Spanish male PE students. While they expressed clear opposition to GBV, they simultaneously regarded aggressive behavior as a normative aspect of sport culture. Traits such as dominance, strength, and determination were seen as integral to athletic performance, though not exclusive to male athletes. PE students emphasized that both women and men could display aggression and use sport as a channel for emotional release. From their perspective, aggressive behavior could act as a performance enhancer, motivating athletes to excel. However, this belief may complicate GBV prevention efforts, as it risks normalizing violent behavior within competitive, performance-driven environments.

I believe that aggressiveness does not necessarily equate to violence, so I do not see it as inherently negative. Boys can be aggressive—not that they must be, but they sometimes are. And the same goes for girls. Aggressiveness, as I see it, can manifest as determination or strength, a drive to pursue goals (PE student Spain).

### 3.3 Strategies employed by PE students when acting as bystanders during incidents of GBV

The strategies employed by PE students in response to GBV could be grouped in four categories: positive and negative proactive bystander behavior, and positive and negative reactive bystander behavior (16). Positive proactive and negative reactive behaviors were particularly evident in students' communication patterns. In response to subtle or less overt instances of GBV, such as remarks like “not bad for a girl”, many students expressed a desire to challenge gender stereotypes and promote more open dialogue, reflecting positive proactive bystander behavior. This attitude was especially prominent in discussions concerning gender expectations in sport, particularly football. Female PE students shared that during football practices, they were frequently overlooked by male peers, who passed the ball more often to other boys. As a result, female students had fewer opportunities to participate fully, limiting their chances to develop skills and be more successful in the sport.

The boys get way more passes, and it is kind of crappy because we [girls] are just left standing there. I know I am not super good at ball sports, I am aware of that. But if I never get to join in and practice, I cannot improve either (PE student Belgium).

It has already happened before. Someone makes a great shot, and you hear: ‘Not bad for a girl.’ But no, it is not because I am a girl. I can perform just as well as the boys, and that does not change anything. I would respond by saying: ‘Well, there you go, girls are good at team sports too,’ or something along those lines (PE student Belgium).

Positive reactive bystander behavior was most commonly described in situations involving mild verbal aggression and instances of sexual violence, although strategies varied depending on the context. In response to mild verbal violence, students reported being more inclined to intervene when the perpetrator was a peer. Strategies included asking individuals to clarify their comment or pointing out the potential harm caused by their words. In contrast, in situations involving sexual violence, students emphasized the importance of prioritizing the victim's safety—typically by removing them from the situation—and then offering support based on the victim's needs. Suggested forms of support included remaining physically close to the victim or making eye contact to convey reassurance.

Reactions to physical violence were more diverse. While there was general agreement on the need to intervene, approaches ranged from involving authorities, for example professors, coaches, or the police, to seeking help from other bystanders. Some male students admitted to considering physical violence as retaliation against the perpetrator, though only if they felt confident they could safely leave the situation afterward, reflecting negative reactive bystander behavior.

A notable concern was the widespread lack of awareness regarding official reporting channels for harassment and abuse within their HEIs and sports clubs. Only one participant knew the safeguarding officer in their club. In addition, students expressed limited trust in the effectiveness and transparency of existing reporting channels. Many were unclear about their HEIs procedures, uncertain whether reports would be taken seriously, and skeptical about the likelihood of consequences for perpetrators. While students acknowledged the value of having designated support services, they also voiced doubts and concerns about the neutrality of safeguarding officers. This perceived lack of trust, clarity, and effectiveness may act as a barrier to official reporting and seeking help.

The accessibility of support services is a concern. Personally, I find it really difficult to figure out where to turn for help. At my HEI in particular, I find the process challenging. For example, the website might direct you to one place, but then you are referred to somewhere else, and ultimately, you must wait another three months. There should be a system that allows for quick access, especially when something urgent or serious has happened. If you're forced to wait three months, it might not even feel worthwhile anymore. Imagine something happens tomorrow, and you want to speak to a professional about it. By the time those three months pass, you have already spent so much time overthinking it, and you might even start to believe ‘Maybe it was my fault.’ That kind of thought is incredibly difficult to shake. But if you could access help the very next day, it could make a huge difference (PE student Belgium).

### 3.4 Perceived barriers influencing student's positive reactive bystander behavior

PE students identified three key barriers that hinder positive bystander behavior in situations of GBV. The first barrier was uncertainty regarding the relationship between those involved, for example not knowing whether they are friends or romantic partners. This ambiguity often led to inaction, as students hesitated to intervene without knowing the social norms within that relationship or context, including the use of dark humor or flirtatious behavior. The second barrier concerned the hierarchical position of the perpetrator. When inappropriate behavior originated from someone in a position of authority, such as academic staff or coaches, students were generally reluctant to respond. They were concerned that intervening could negatively affect their academic standing and future grading by that individual. A small number of students in both countries, however, reported that they would intervene regardless of hierarchy, guided by personal values that compelled them to speak out.

The third barrier was the individual stress response when witnessing GBV. Although many students expressed a willingness to intervene, they also acknowledged tendencies to freeze or withdraw in real life situations. Two primary factors contributed to this reaction. Firstly, fear of negative consequences. Students feared becoming targets themselves, particularly in cases of sexual or physical violence. Female students, in particular, voiced concerns about becoming victims if they stepped in. Others, regardless of gender, worried about being physically harmed. Secondly, a perceived lack of competence. Many PE students felt unsure about the appropriate steps to take and doubted their ability to intervene effectively. In such cases, inaction appeared to be the safer choice. However, students noted that knowing either the victim or the perpetrator, or having a trusted individual nearby, increased their confidence and likelihood to act.

If I actually see someone using physical violence, I completely freeze. I cannot do anything at all. So it could very well happen that I am standing just ten meters away, completely still, unable to do anything…[explains a situation they witnessed as a bystander]… Looking back, it was hard for me to come to terms with the fact that I did not do anything, but you never know how you are going to react because it could be different next time. At that moment, it was so overwhelming for me to see it happening right in front of me that I thought, wow, this is really happening right now, and I just completely froze (PE student Belgium).

## 4 Discussion

This study sought to examine undergraduate PE students' knowledge, attitudes, and intended behaviors concerning GBV within the contexts of sports and HEIs. The findings of the study revealed three key insights. Firstly, there is a difference in interpretation of GBV across countries. Spanish PE students, in comparison to the Belgian students, use a binary perspective of GBV. Meaning they perceive GBV as all violent acts against women perpetrated by men. However, it is imperative that GBV prevention interventions move away from the traditional discourse of women as victims and men as perpetrators and adopt a more progressive perspective on the concept of gender and GBV (4). Globally, women still experience disproportionately more violence compared to men (25). However, when looking at evidence from the sport sector, this statement is less conclusive. 5 Hartill et al. (2022) discovered that male athletes, in comparison to female athletes, reported higher levels of interpersonal violence before the age of 18. Nevertheless, the perceived cause of this violence often remains unknown. Thus, we cannot confirm or refute the lived experiences of GBV among men. Research, however, is unanimous in concluding that LGBTQIA+ individuals are at a higher risk for violence (8, 9, 26, 27). Thus, it could be argued that individuals who deviate from the prevailing gender identity, gender expression, or sexual orientation within the sport context are at risk of experiencing GBV, thus including men. A progressive perspective on GBV would allow to recognize men's lived experiences with GBV, as well as recognizing the nefast gender norms, and gender stereotypes that facilitate the perpetration of GBV.

Secondly, aggression was seen as inherent to the sport context by Spanish PE students and, when used intentionally to gain a competitive advantage, it has been identified as instrumental interpersonal violence (28). Yet, PE students did not attribute the aggressive behavior to only men. On the contrary, the gender stereotype of “women are weak” was reputed by the students, however, not the patriarchal and macho culture where violent behavior is tolerated and expected (29), and wherein GBV could be used to gain an advantage. By generalizing violent behavior to all genders, it may shift the female gender stereotypes, but the social norm of using and accepting intentional violent behavior in sport remains. These findings support the claim that the prevention of GBV starts at the quality level of an organization. 27 Tuakli-Wosornu et al. (2024) illustrated that interpersonal violence can occur at the interpersonal level, organizational level, and societal level. Therefore, bystander GBV prevention intervention should include the influence of negative and positive social norms, gender norms, and gender stereotypes on GBV at multiple levels, as they may influence each other. Furthermore, bystander interventions should also include competence trainings, such as communication skills, to support a safe and inclusive sport climate.

Thirdly, this study highlights the importance of recognizing the natural human stress response of bystanders. PE students demonstrated the necessary knowledge to understand a GBV situation, felt responsible to act, and implemented positive reactive bystander behavior strategies, such as taking the victim out of a situation, addressing the perpetrator, or calling for help (17). However, they acknowledged that their perceived behavioral control to engage in positive bystander behavior was low due to the anticipated stress they would experience when witnessing GBV. This shows that even with the best behavioral intentions, achieving positive reactive bystander behavior would depend on the experienced stress levels of the bystander when witnessing or encountering GBV.

To date, the natural human stress response to witnessing GBV is not sufficiently integrated in existing bystander prevention interventions. However, when humans are faced with danger, their parasympathetic nervous system shuts down, and their sympathetic nervous system sends the signal that danger is near. If stress levels are high enough, individuals rely on their survival instincts to act, resulting in freeze, fight, or flight reactions (24). Yet, these natural responses are often categorized in bystander literature as negative bystander behaviors, such as inaction, walking away, or retaliatory violence (30). Even though individuals act on instinct and thus do not actively choose their behavior. The reason why bystanders do not portray the expected desired behavior is not always due to a lack of awareness or knowledge on intervention strategies, but rather a lack of self-regulation and crisis management skills to achieve the desired outcomes.

GBV prevention interventions primarily emphasize knowledge transfer and foster attitude changes at the individual level (14, 31). For instance, many programs incorporate intervention strategies as key components. However, most interventions focus solely on teaching “what to do” and often neglect the critical aspect of “how to do it” (31). To promote effective bystander behavior, GBV prevention initiatives should not only disseminate knowledge but also cultivate essential skills, such as stress management, that can empower bystanders to take positive action. An experiential learning approach could therefore be an appropriate methodology for PE students, as it stimulates action (32). Overall, a holistic approach should be adopted, by paying attention to knowledge, social skills, and self-regulation, in the context of preventing GBV in sport.

### 4.1 Strengths, limitations and future research directions

A first strength of this study was the direct access to undergraduate PE students, which enabled the collection of valuable insights into the prevention of GBV from their perspectives and experiences. A second strength is its cross-cultural scope. By comparing the perspectives of undergraduate PE students in Spain and Belgium, the study reveals important differences in how GBV is interpreted across countries. The study challenges the traditional perspective and underscores the need for more inclusive and progressive GBV prevention interventions. This contributes to the global discourse on GBV by broadening the understanding of how gender norms and identities intersect with violence in sport. A third strength of the study is its focus on the often-overlooked human stress response in bystander behavior. The study effectively highlights the role of the nervous system—specifically the freeze, fight, or flight response—in shaping bystander actions. This insight shifts the focus from a purely cognitive model of intervention to one that incorporates physiological and emotional regulation skills, adding an important dimension to existing bystander intervention literature.

The study encountered several limitations. The study was part of a larger EU co-funded project led by organizations from Belgium and Spain, and the data was collected in three different languages (i.e., French, Dutch and Spanish). The multiple languages made the adherence to research standards challenging, particularly in the translation of the online survey and interview transcripts from Dutch to English to Spanish. In the absence of a Spanish-speaking researcher, the Spanish transcripts had to be translated to English, without the possibility of conducting back-translation to verify whether the nuances of participants' responses were accurately captured. International research often involves multiple languages and resources for translations or linguistic expertise are often limited, potentially affecting the accuracy of translated transcripts. Moreover, participation by the PE students was voluntary, which may have led to a selection bias. It is possible that the data reflects the knowledge, attitudes, and intended behaviors of PE students already convinced of the importance of preventing GBV and therefore more inclined to participate in the study.

This study utilized the theory of planned behavior (18) and the stages of bystander theory (17) to conceptualize (intentional) positive bystander behavior. Both theoretical frameworks offer explanations for bystander behavior using cognitive perspectives (i.e., knowledge on GBV or norms and beliefs on GBV). However, witnessing an incident of GBV can evoke a stressful response, potentially inhibiting the rational processes of the brain (24). The findings of this study suggest that existing bystander theories fail to adequately account for the role of the nervous system's response when bystanders witness situations involving GBV (24). Consequently, future research should delve into the impact of acute stress responses (i.e., freeze, fight, or flight) to better understand how stress affects bystanders' ability to adequately intervene in a GBV situation. Additionally, there is a pressing need to document the lived experiences of male and LGBTQIA+ survivors of GBV, as well as questioning perpetrators on their reasoning behind the use of violence, particularly in specific settings such as sports and HEIs, to address gaps in understanding why violence manifests.

## 5 Conclusion

This study provides valuable insights into the perceptions, attitudes, and intentional behaviors of undergraduate PE students regarding GBV prevention in sports and HEIs. Notably, it highlights a critical gap in current bystander behavior research, namely the failure to acknowledge the natural human stress response when witnessing a GBV situation. Furthermore, the knowledge and trust in the procedure to report GBV inside sports organizations or HEIs is lacking. This oversight may partially explain why bystanders often struggle to exhibit the desired behaviors. Consequently, to achieve positive bystander behavior GBV prevention interventions should acknowledge experienced barriers and thus, go beyond knowledge transfer, incorporating comprehensive skills training that equips individuals to respond effectively under perceived pressure.

## Data Availability

The datasets presented in this article are not readily available because sharing the raw or anonymized data (e.g., transcripts) would interfere with the informed consent process, as participants were not explicitly asked for consent to disclose this information. Requests to access the datasets should be directed to tiphaine.clerincx@vub.be.
